# Integrative Analysis of the Invasive Pathways of the Ragweed Leaf Beetle *Ophraella communa* LeSage 1986 (Coleoptera, Chrysomelidae) Into Domestic Areas of the Korean Peninsula

**DOI:** 10.1002/ece3.73876

**Published:** 2026-06-18

**Authors:** Tae Hwa Kang, Sun Jae Park

**Affiliations:** ^1^ Quarantine Technology Institute Inc. Gimcheon‐si Gyeongsangbuk‐do Korea; ^2^ National Institute of Biological Resources Incheon Korea

**Keywords:** exotic species, genetic structure, microsatellites, mitochondrial genes, *Ophraella communa*, spread scenario

## Abstract

The ragweed leaf beetle *Ophraella communa* LeSage is an exotic species reported to have been introduced to Korea in 2002. It has spread and inhabits the entire Korean Peninsula using ragweed, an alien plant that invaded the Korean Peninsula after the Korean War (1950–1953), as its host plant. To infer the spread routes of the exotic ragweed leaf beetle in domestic areas of the Korean Peninsula, we analyzed the genetic structure and demographic history of the species collected from 12 domestic areas leveraging mitochondrial DNA sequence (MT) and microsatellite loci genotype (MS). For MT, haplotype network analysis identified two high‐frequency haplotypes linked via eight median vectors. MS indicated that the species had three invasive routes into the Korean Peninsula and was divided into three groups. MT and MS results differed in the number of invading populations considered. These differences were suggested to stem from resolution variations due to the founder effect. In conclusion, at least two *O. communa* populations may invade several international ports of the Korean Peninsula and spread domestically via hitchhiking on transport. Individuals spread by transportation may disperse into local areas due to the behavioral traits of *O. communa* being strongly attracted to the host plant *Ambrosia artemisiifolia* . We anticipate that our findings will help pinpoint critical areas for preventing the inland spread of exotic species.

## Graphical abstract

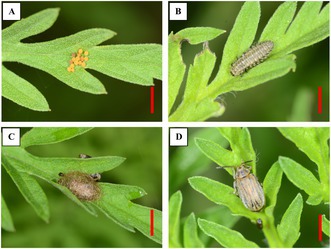

## 1 Introduction

Among *Ambrosia* spp., ragweed ( *A. artemisiifolia* ) known as a noxious agricultural weed has become invasive and spread worldwide, often replacing native plants by thriving in large numbers on roadsides, railroad tracks, construction sites, cultivated lands, and other disturbed ecosystems (Bosio et al. 2014; Makra et al. 2015; Montagnani et al. 2017; Lee et al. 2021). In cultivated lands, ragweed effectively utilizes large amounts of fertilizer, have high productivity, and regenerate well in dry and infertile soils. Ability of the invasive plant to block sunlight by rapid growth causes reduced crop productivity (Xie et al. 2001; Makra et al. 2015). In addition, ragweed produces allergens that affect humans. Male flowers produce large amounts of wind‐borne pollen, which causes hay fever, rhinoconjunctivitis, asthma, and skin irritation (Wopfner et al. 2005; EFSA 2010; Bosio et al. 2014; Yoon et al. 2014; Bonini et al. 2022). In Korea, ragweed was first recorded on the Korean Peninsula in 1968; however, its introduction is believed to have occurred around the time of the Korean War (1950–1953) (Lee 1968; S. H. Park 1995; Kim et al. 2017). Ragweed has a high growth‐inhibiting effect on other plants, inhibiting the germination and early growth of native plants and crops on the Korean Peninsula. Therefore, the Ministry of Environment of Korea designated ragweed as an “alien plant harmful to the native ecosystem” in 1999 (Choi et al. 2010; Huh and Kim 2023).

Due to human health problems associated with ragweed, several countries, including China, Japan, and Australia, have conducted research on the use of ragweed leaf beetles as a biological control agent for ragweed (Yamazaki et al. 2000; Palmer et al. 2010; Zhou et al. 2010, 2014; Guo et al. 2011). In Europe, research has been conducted on the biocontrol potential of ragweed leaf beetle to mitigate ragweed crop damage (Kovalev et al. 1983; Müller‐Schärer et al. 2014). The results confirmed that ragweed leaf beetles primarily feed on ragweed. However, some individuals complete their life cycles on cultivated crops, including sunflower ( *Helianthus annuus* ) (Futuyma and McCaffery 1990; Palmer and Goeden 1991; Emura 2000). In Australia, the ragweed leaf beetle is listed as a prohibited insect pest due to its potential to cause crop damage (Palmer and Goeden 1991). In recent studies in Canada and China, *O. communa* rarely lays eggs on sunflower under choice condition, larval survival on sunflower is low, and newly emerged adults leave the sunflower plants in search of *A. artemisiifolia* (Dernovici et al. 2006; Cao et al. 2011; Zhou et al. 2011; reviewed in Müller‐Schärer et al. 2014).

**FIGURE 1:**
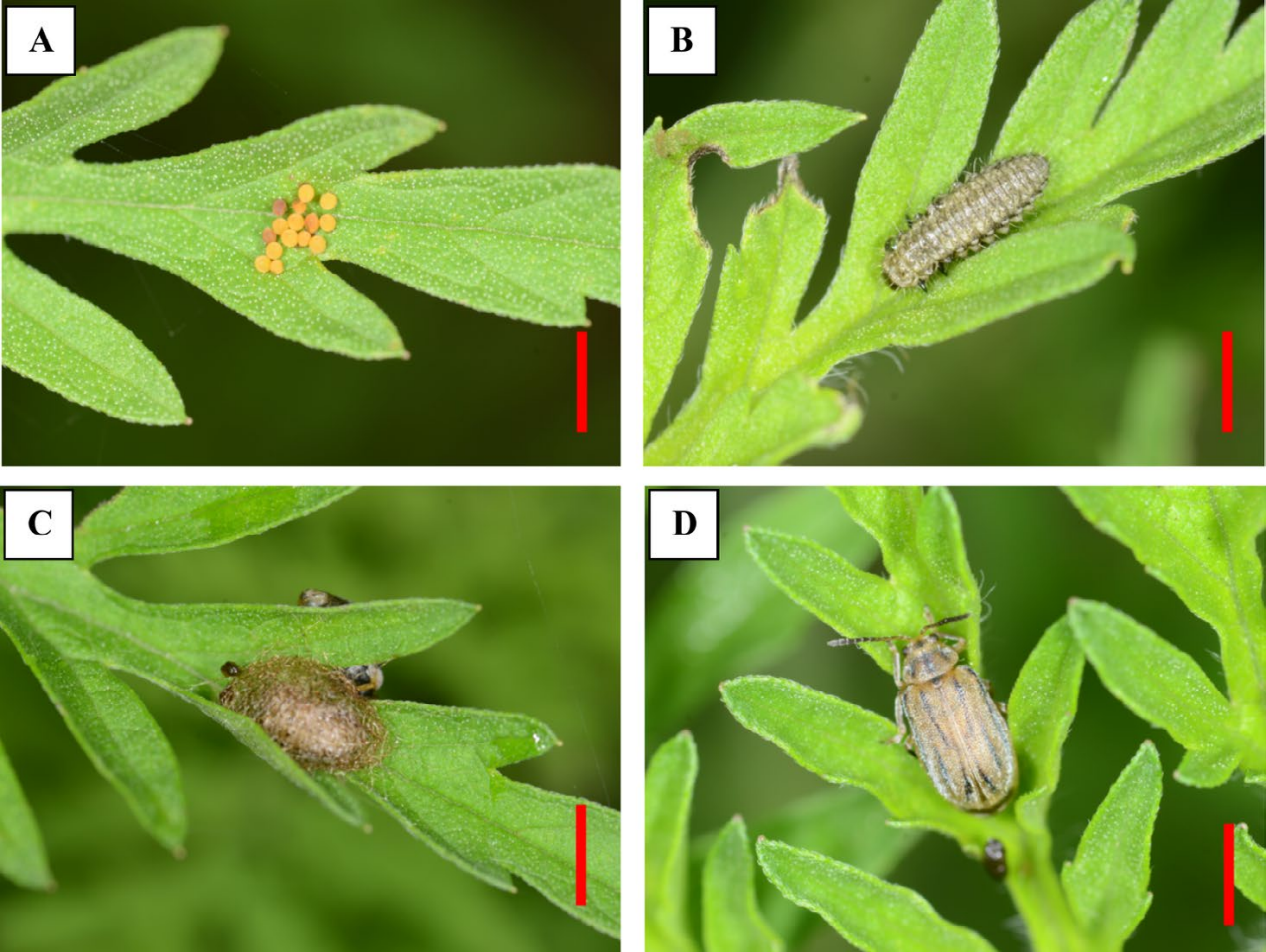
Habitus of each life stage of *Ophraella communa* on *Ambrosia artemisiifolia* in the wild. (A) Egg mass; (B) larva; (C) pupae; (D) adult (Scale bar = 5.0 mm).

The ragweed leaf beetle *Ophraella communa* LeSage (1986) (Chrysomelidae, Coleoptera), originating from North America, is an internationally spread exotic species that has been introduced to Asia (China, Taiwan, and Japan in 1996; Korea in 2000; India, Malaysia, and Singapore in 2021) and Europe (Italy and Switzerland in 2013; Slovenia in 2017; Croatia in 2018; Bosnia, Serbia, Hungary, and Romania in 2020) (Wang and Chiang 1998; Takizawa et al. 1999; Sohn et al. 2002; Boriani et al. 2013; Müller‐Schärer et al. 2014; Seljak et al. 2017; Zadravec et al. 2019; Karrer et al. 2020; Petrović‐Obradović et al. 2020; Horváth and Lukátsi 2020; Keszthelyi et al. 2023). The ragweed leaf beetle is an oligophagous species that may feed on 18 plant species spanning 4 families, including 15 species in Asteraceae, 1 in Fabaceae, 1 in Euphorbiaceae, and 1 in Cannabaceae; among these host plants, the main host is *Ambrosia* spp., spending its entire life cycle on the host plant (Palmer and Goeden 1991; Kim et al. 2017; Lee et al. 2023; Rousset et al. 2024) (Figure 1).

In Korea, the ragweed leaf beetle was first recorded in Hwawon Park, Dalseong‐gu, Daegu‐si, in 2000 (Kwon et al. 2001; Sohn et al. 2002). Thereafter, it was reported to occur in riverside areas in Daegu‐si and the demilitarized zone in Gyeonggi‐do (Kwon et al. 2001, 2001a, 2001b). Although the exact spread routes have not been identified, in 2001, the occurrence of the beetle was confirmed throughout Korea, and it was estimated that it had spread throughout the Korean Peninsula before 2000 (Sohn et al. 2002). In 2010, the species dispersed to Jeju Island and is now confirmed to be distributed throughout the country, including the island region (Kim et al. 2017).

For biological control on ragweed listed as an alien plant harmful to the native ecosystem in the Ministry of Environment of Korea, research on the possibility of using ragweed beetles as biological control agents is being conducted in Korea, and the beetle is being confirmed to be effective in controlling ragweed (Kim et al. 2017; Lee et al. 2023). However, additional research is needed, as a field survey has revealed that ragweed leaf beetles feed on the horticultural plant *Rudbeckia bicolor* , and thus, host shifting of the ragweed beetle population in Korea is suspected (Park et al. 2009). Therefore, rather than using the ragweed leaf beetles currently widespread and established on the Korean Peninsula as a means of ragweed control in their wild state, it is necessary to utilize them under controlled conditions through population management such as the spread prevention monitoring on *A. artemisiifolia* and *O. communa* .

In this study, we attempted to infer the spread routes of the invasive population of ragweed leaf beetles on the Korean Peninsula using population genetic analysis based on mitochondrial genes and microsatellite loci, focusing on beetles that are widespread and established within the native ecosystem invaded by ragweed. Inferring the introduction history from genetic markers is important for elucidating the patterns of colonization and expansion of introduced species and biocontrol agents (Estoup and Guillemaud 2010; Nishide et al. 2015). Therefore, this study aimed to analyze the genetic diversity of ragweed leaf beetles inhabiting the Korean Peninsula, analyze the genetic structure among local ragweed leaf populations, and estimate the route of spread of the ragweed beetle within the Korean Peninsula.

## 2 Materials and Methods

### 2.1 Sampling and Genomic DNA Extraction

**FIGURE 2:**
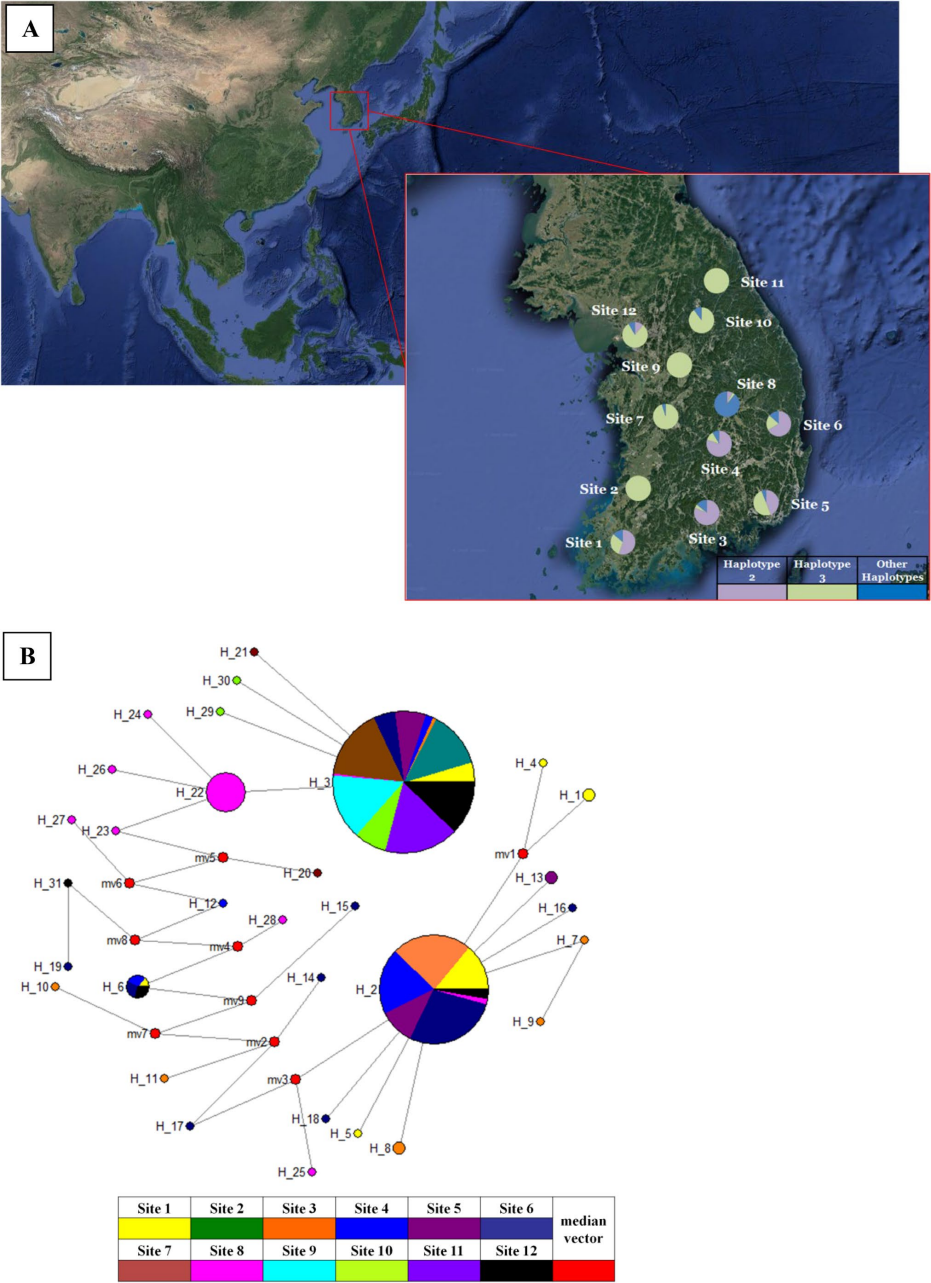
Mitochondrial gene‐based genetic structure of *Ophraella communa* that has invaded Korea. (A) Haplotype composition of each regional population; (B) Median‐joining network (Satellite map ver. 2015 provided by National Geographic Information Institute under Ministry of Land, Infrastructure and Transport of Korea).

For selection of collecting points of *O. communa* , we, we firstly divided the Korean Penninsula into 12 regions along the Korean administrative districts. The collecting point of each region was randomly selected considering distance among each point (Table 1; Figure 2A). Sampling was carried out from June to August, when it was known as *O. communa* main occurrence period (Sohn et al. 2002; Kim et al. 2017). Around the randomly selected point of each region, we searched open areas of river or waterway side, where there might be like growing, and carefully seek in a 500 m radius. Three to five adult individuals per head of ragweed were collected using an insect poison bottle containing 70% ethyl alcohol. In the laboratory, the collected beetles were morphologically re‐examined based on Sohn et al. (2002) under a stereo microscope, Leica MS 5 (Leica, Wetzlar, Germany). The identified samples were washed with 100% ethyl alcohol and stored in eppendorf tubes by each individual under −20°C. The samples for next‐generation sequencing (NGS) were separately collected constructing the reference sequence library on *O. communa* . Sample collecting and storing methods were the same as above, but the collecting point did not overlap with that for genetic structure analyses (Table 1), and the samples in a bottle containing ethyl alcohol were stored at −20°C. The genomic DNA (gDNA) of the collected samples was extracted using a DNeasy Blood & Tissue Kit (Qiagen, Hilden, Germany) according to the manufacturer's instructions. The gDNA for genetic structure analyses was extracted by each individual, but that for constructing the reference sequence library was from pooled 10 individuals. The gDNAs were stored under −20°C.

**TABLE 1:** Collection and sequencing information of *Ophraella communa* in Korea.

SN	Collecting location	GPS	CD	CSN	SSN	GBAN			GSN
						COI	ND5	ATP8/ATP6	
1	JN Yeongam‐gun Gunseo‐myeon Dogap‐ri Around Temple Dogapsa	34°45′11″ N 126°39′39″ E	25. VI. 2020.	36	36	36 (OL690593–OL690628)	36 (OL754676–OL754711)	36 (OL755121–OL755156)	20
2	JB Jeongeup‐si Ssangam‐dong Around Mt. Naejangsan	35°30′27″ N 126°54′22″ E	25. VI. 2020.	31	31	31 (OL690629–OL690659)	31 (OL754712–OL754742)	31 (OL755157–OL755187)	20
3	GN Jinju‐si Daepyeong‐myeon Daepyeong‐ri	35°13′24″ N 127°57′12″ E	02. VII. 2020.	48	42	45 (OL690660–OL690704)	44 (OL754743–OL754786)	48 (OL755188–OL755235)	20
4	GB Gimcheon‐si Yulgok‐dong Around Yongjeongyo	36°07′38″ N 128°10′41″ E	02. VII. 2020.	35	35	35 (OL690705–OL690739)	35 (OL754787–OL754821)	35 (OL755236–OL755270)	20
5	Busan‐si Saha‐gu Hadan‐dong	35°06′25″ N 128°56′54″ E	09. VII. 2020.	34	34	34 (OL690740–OL690773)	34 (OL754822–OL754855)	34 (OL755271–OL755304)	20
6	GB Cheongsong‐gun Juwangsan‐myeon Ji‐ri Aroung Bridge Mapyeonggyo	36°22′24″ N 129°05′37″ E	16. VII. 2020.	61	60	61 (OL690774–OL690834)	60 (OL754856–OL754915)	61 (OL755305–OL755365)	20
7	CN Yeongi‐gun Seo‐myeon Dosingobok‐ro Around Gobok Lake	36°30′30″ N 127°14′55″ E	08. VII. 2020.	42	41	42 (OL690835–OL690876)	41 (OL754916–OL754956)	42 (OL755366–OL755407)	20
8	GB Sangju‐si Donam‐dong Around Nakdonggang National Institute of Biological Resources	36°26′35″ N 128°15′21″ E	15. VII. 2020.	30	28	30 (OL690877–OL690906)	28 (OL754957–OL754984)	30 (OL755408–OL755437)	20
9	GG Icheon‐si Moga‐myeon Singal‐ri Around Bridge Singalgyo	37°12′26″ N 127°27′00″ E	13. VIII. 2020.	40	36	40 (OL690907–OL690946)	36 (OL754985–OL755020)	40 (OL755438–OL755477)	20
10	GW Chuncheon‐si Sanong‐dong Is. Gogumaseom	37°54′58″ N 127°43′07″ E	16. VII. 2020.	22	20	22 (OL690947–OL690968)	22 (OL755021–OL755042)	20 (OL755478–OL755497)	20
11	GW Yanggu‐gun Nam‐myeon Gaojak‐ri Around Gwangchi Valley	38°07′51″ N 128°03′23″ E	16. VII. 2020.	49	41	49 (OL690969–OL691017)	42 (OL755043–OL755084)	48 (OL755498–OL755545)	20
12	Incheon‐si Seo‐gu Oryu‐dong Around Road Arabaetgil	37°34′12″ N 126°38′27″ E	24. VIII. 2020.	39	36	39 (OL691018–OL691056)	36 (OL755085–OL755120)	39 (OL755546–OL755584)	20
A	JN Jangseong‐gun Bukha‐myeon Sangung‐ri Around Lake Jangseongho	35°25′15″ N 126°50′51″ E	17. VII. 2020.	20	For NGS	—	—	—	—
Total	13 sites			487	440	464	445	464	240

Abbreviations: ATP6, adenosine tri‐phosphate synthase 6; ATP8, adenosine tri‐phosphate synthase 8; CD, collecting date; COI, cytochrome c oxidase I; CSN, number of collected samples; GBAN, GenBank accession no.; GSN, number of genotyping sample; ND5, nicotinamide adenine dinucleotide dehydrogenase 5; SN, site no.; SSN, number of sequenced samples.

### 2.2 Mitochondrial Analysis

#### 2.2.1 Mitochondrial DNA Marker Development and MT Gene Sequencing

Genetic markers for the haplotype analysis of the MT DNA of ragweed leaf beetles were designed using the mitochondrial genome sequence obtained from GenBank (https://www.ncbi.nlm.nih.gov/genbank/) (KY039100 by Song et al. 2017). Four mitochondrial genes were identified: *cytochrome c oxidase I* (COI), *nicotinamide adenine dinucleotide dehydrogenase 5* (NADH5), *adenosine tri‐phosphate synthase 8* (ATP8), and *adenosine tri‐phosphate synthase 6* (ATP6). The genetic markers for each gene were designed using PRIMER3, following the criteria of a 20‐mer primer length and melting temperature (*T*_m_) of 50°C (Untergasser et al. 2012). ATP8 and ATP6 are adjacent genes; therefore, we designed a single primer set from the ATP8–ATP6 gene complex containing the latter part of ATP8 and the former part of ATP6. The length of the target sequence for each gene was 692 bp for COI, 609 bp for NADH5, and 619 bp for ATP8–ATP6 (Table 2).

**TABLE 2:** Primer design for target mitochondrial genes of *Ophraella communa* .

No.	Target gene	Primer name	Primer sequences	Target gene size	Reference sequence
1	COI	COI_F	TTTATTTTTGGTATTTGAGC	692 bp	KY039100 (Song et al. 2017)
		COI_R	TCTTCTTTCCTGTCTTACAA		
2	NADH5	ND5_F	TGATGAATATATAGCTGGAGA	609 bp	
		ND5_R	GATAATTACCGACTGCTAAA		
3	ATP8‐ATP6	ATP_F	TTCCACAAATAATACCCTTA	619 bp	
		ATP_R	ATAAGTGACCTGCAATTATG		

To amplify MT genes of each local population, we used AccuPower PCR Premix (Bioneer, Daejeon, Korea) in a total reaction volume of 20 μL comprising 1 μL gDNA, 1 μL forward primer (10 pmol/μL), 1 μL reverse primer (10 pmol/μL), and 17 μL distilled water. Amplification for each marker was conducted by using a PCR Thermal Cycler Dice Touch (TaKaRa Bio Inc., Shiga, Japan) under the following conditions: initial denaturation at 94°C for 5 min (1 cycle); amplification at 94°C for 10 s, 50°C for 10 s, and 72°C for 20 s (30 cycles); and a final extension at 72°C for 5 min (1 cycle). Thereafter, the PCR products were sequenced in both directions using a 3739xl DNA analyzer (Thermo Fisher Scientific, Waltham, MA, USA).

#### 2.2.2 Mitochondrial Sequences Data Analysis

Raw sequence data for each gene from each sample were aligned and trimmed using MEGA7 (Kumar et al. 2016), and the trimmed sequences were submitted to GenBank (https://www.ncbi.nlm.nih.gov/genbank/) (Table 1). The trimmed sequences from each genetic marker were made as one concatenated dataset for population genetic analyses. The number of haplotypes and polymorphic sites was estimated using DnaSP ver. 5.10 (Librado and Rozas 2009) based on the aligned sequence dataset, which was then converted into datasets for Arlequin and NETWORK. Molecular diversity indexes (haplotype and nucleotide diversity) were estimated using a pairwise difference model in Arlequin ver. 3.1 (Excoffier et al. 2005). To estimate the genealogical relationships among haplotypes, a median‐joining network was constructed using NETWORK ver. 10.2.0.0 (https://www.fluxus‐engineering.com/index.htm). The haplotype composition graph at each local site was calculated by using MS‐Excel (Microsoft, Redmond, DC, USA), focusing on the high‐frequency haplotypes H_2_ and H_3_. The obtained graph was mapped in each collecting site (Figure 2A). To estimate genetic differentiation among regional populations, an analysis of molecular variance (AMOVA) was performed using a pairwise difference model with 1000 permutations in Arlequin ver. 3.1. The *F*_ST_ distances among all pairs in the population were used to assess the genetic structure of ragweed leaf beetles that invaded Korea. Pairwise population *F*_ST_ values were calculated using the pairwise difference model in Arlequin ver. 3.1 (significance test = 0.05; significance level = 1000 permutations). Based on pairwise genetic distances, a network estimating the genealogical relationships among the 12 regional populations was constructed using the ordinary least squares algorithm in SplitTree4 ver. 4.14.4 (Huson and Bryant 2006).

### 2.3 Microsatellite Analysis

#### 2.3.1 Next‐Generation Sequencing

The obtained gDNA was stored at −20°C, after which NGS was conducted using an MGISE‐Q2000 platform (MGI Tech, Shenzhen, China) at GenCube Plus (Seoul, Korea). For assembly, sequencing errors were discarded using the error correction module of SOAPec ver.2.02 (Luo et al. 2012). After error correction, genome assembly was performed using SOAPdenovo2 ver. 2.04‐r240 (Luo et al. 2012). To identify reliable assemblies, short reads were remapped to assembled sequences using GapCloser ver 1.12 (Luo et al. 2012) and only assembled scaffolds were retained for microsatellite marker identification.

#### 2.3.2 Microsatellite Locus Identification, Marker Development, and Genotyping

Microsatellite loci searching from assembled contigs through NGS was conducted using MISA (Beier et al. 2017), and primer sets for identified microsatellite loci were designed using PRIMER3, following the criteria of an 18–22‐mer primer length and *T*_m_ of 50°C–55°C (Untergasser et al. 2012). The genetic markers for population structure analysis were first screened using the following criteria: exclusion of AT‐rich microsatellite loci; selection of loci with 2–4 nucleotide repeats; exclusion of sequences unsuitable for primer design; selection of markers with a 20‐mer length and *T*_m_ of 52°C; selection of microsatellite loci with 15–25 nucleotide repeats; selection of PCR products with a size of 150–270 bp; and exclusion of duplicate sequences. We selected genetic markers with high PCR efficiency through electrophoresis using agarose gel on PCR products amplified with non‐dye primer sets. The forward primers of the final selected markers (Table 3) were labeled with 6‐carboxyfluorescein at the 5′ end (Schuelke 2000).

**TABLE 3:** The nine developed markers for microsatellite loci analysis of *Ophraella communa* populations that have invaded Korea.

No.	Microsatellite loci No.	Sequences of primer set	Repeat motif	Target sequence size	Remarks
1	101,695	GAAGAACTATTAAGCACTGC	(AAG)_17_	253	Selected
		GATCAGATCATCCAATCACA			
2	9248	TATAGGTCGCAGTGTACTTA	(CATA)_21_	205	Selected
		GCGATTGGTAAGAGTCTATT			
3	2,689,147	ACTATCCTTCTATCCGATCG	(AG)_21_	156	Selected
		TACAATTGGATAGTAACCGC			
4	1,571,517	CATCTCGTATAGTAGCAGTT	(CA)_21_	177	Selected
		CCATCTAACTATCTGTGACA			
5	558,867	GTCAACACTCAACACTAGAT	(CTT)_17_	213	
		GCATCAATACTTACCGGATA			
6	580,013	GATCGACCACTTAATGTTAC	(GT)_21_	154	Selected
		TATAGTTGTGGTCATTACGC			
7	14,631	ACACCTTGTAGACATGTATG	(GTAT)_20_	191	
		CCGAATTCTTCCTATCAGAT			
8	11,007	CTGAAGAATTGGAAGTTGAG	(TC)_22_	245	Selected
		GACCAATCCGAGTACTTATT			
9	420,873	GATAAGTCACTGAGGATAGC	(TC)_18_	254	Selected
		ATCCTGTAGTACCTCATCTT			

For the PCR of microsatellite loci of each sample, we used Han Taq Polymerase (HanLAB, Cheongju, Korea) in a 20‐μL reaction volume comprising 0.2 μL Han Taq (5 U/μL), 2–3 μL gDNA, 1 μL forward primer (10 pmol/μL), 1 μL reverse primer (10 pmol/μL), 2 μL 10X reaction buffer, 2 μL dNTP mix (10 mM), and 10.8–11.8 μL distilled water. PCR for each genetic marker of each local population was conducted using a PCR Thermal Cycler Dice Touch system (TaKaRa Bio Inc.) under the following conditions: initial denaturation at 94°C for 5 min (1 cycle); amplification at 94°C for 30 s, 49°C for 30 s, and 72°C for 30 s (34 cycles); and final extension at 72°C for 5 min (1 cycle). PCR products were genotyped using a 3739xl DNA analyzer (Thermo Fisher Scientific). The genotyping results for each sample were rearranged using Microsoft Excel (Microsoft Corporation, Redmond, WA, USA) and TextPad (HELIOS, Longridge, UK).

#### 2.3.3 Microsatellite Loci Data Analysis

Genotyping errors such as null alleles and scoring errors on selected markers were checked using Micro‐Checker ver. 2.2.3 (Oosterhout et al. 2004). Genetic diversity parameters such as gene diversity, genotype and allele numbers, observed and expected heterozygosity, exact *p*‐value, squared deviation, and Garza–Williamson index (M‐ratio) were calculated using Arlequin ver. 3.1. Hardy–Weinberg equilibrium (HWE) across loci was estimated after sequential Bonferroni correction (Rice 1989). Evidence of a recent genetic bottleneck at each microsatellite locus was determined using the M‐ratio, which was < 0.7, suggesting that a recent genetic bottleneck occurred (Garza and Williamson 2001). Genetic differences among regional populations were determined using AMOVA, which was calculated using the number of different alleles (*F*_ST_ model) with 1000 permutations in Arlequin ver. 3.1. To compute the genetic distances among regional populations, we used pairwise *F*_ST_, which was calculated using the number of different alleles (*F*_ST_ model) with 1000 permutations and a 0.05 significance level in Arlequin ver. 3.1. Based on pairwise genetic distances, a network estimating the genealogical relationships among the 12 regional populations was constructed using the ordinary least squares algorithm in SplitTree4 ver. 4.14.4 (Huson and Bryant 2006). Genetic differentiation among regional populations was tested using model‐based Bayesian analysis in STRUCTURE ver. 2.3.4 (Pritchard et al. 2000; Falush et al. 2003) under the following conditions: an admixture model with correlated‐allele frequencies with a 500,000 burn‐in period, 750,000 MCMC reps after burn‐in, *K* from 1 to 8, and 20 iterations. An admixture model with correlated allele frequencies and sampling location as prior may allow a more accurate detection of genetic structure (Hubisz et al. 2009). Subsequently, the ad hoc statistics Δ(*K*) and mean LnP(*K*) were estimated using StructureSelector (Li and Liu 2018), with the average LnP(*D*) value used to determine the number of genetic groups (Evanno et al. 2005).

## 3 Results

### 3.1 Population Structure Using Mitochondrial DNA

#### 3.1.1 Mitochondrial DNA Sequence Variation

The genetic diversity of *O. communa* introduced to the Korean Peninsula was estimated using mitochondrial DNA sequences. The COI, NADH5, ATP6, and ATP8 sequences of the ragweed leaf beetle were obtained from 440 of 467 individuals collected from 12 local sites on the Korean Peninsula, and the number of individuals analyzed per local site ranged from 20 to 60 (Table 1). Sequence divergence from the MT gene dataset ranged from 0% to 3.3%, and 31 haplotypes were distinguished across 72 polymorphic sites. The gene diversity of the 440 ragweed beetle individuals was 0.5934 ± 0.0172, and among local sites, Site 1 showed the highest value at 0.6095 ± 0.0639, whereas Site 2, 9, and 11 exhibited the lowest value at 0.0000 ± 0.0000 (Table 4). The nucleotide diversity of the 440 individuals was 0.369685 ± 0.179523, with Site 5 showing the highest value at 0.402258 ± 0.200521 and Sites 2, 9, and 11 the lowest value at 0.000000 ± 0.000000 (Table 4).

**TABLE 4:** Genetic diversity indexes for mitochondrial DNA sequences of each regional population.

SN	SEN	HN	GD	ND
1	36	6	0.6095 ± 0.0639	0.351609 ± 0.175583
2	31	1	0.0000 ± 0.0000	0.000000 ± 0.000000
3	42	7	0.3461 ± 0.0940	0.094028 ± 0.050205
4	35	4	0.3529 ± 0.0973	0.211531 ± 0.107658
5	34	3	0.5686 ± 0.0402	0.402258 ± 0.200521
6	60	9	0.5215 ± 0.0673	0.289446 ± 0.143701
7	41	3	0.0963 ± 0.0624	0.006775 ± 0.006608
8	28	9	0.5450 ± 0.1130	0.199442 ± 0.102571
9	36	1	0.0000 ± 0.0000	0.000000 ± 0.000000
10	20	3	0.1947 ± 0.1145	0.005556 ± 0.006005
11	41	1	0.0000 ± 0.0000	0.000000 ± 0.000000
12	36	4	0.3444 ± 0.0959	0.206371 ± 0.105060
Total	440	31	0.5934 ± 0.0172	0.369685 ± 0.179523

Abbreviations: GD, gene diversity; HN, number of haplotype; ND, nucleotide diversity; SEN, number of sequences; SN, site number.

#### 3.1.2 Mitochondrial Genealogy

The genetic structure of ragweed leaf beetle populations introduced to the Korean Peninsula was analyzed based on MT DNA sequences. The median‐joining network constructed using the MT multi‐gene sequences of the 440 ragweed beetle individuals from the Korean Peninsula revealed two high‐frequency haplotypes (H_2_, 143 and H_3_, 241 individuals) connected by eight median vectors (unobserved sequences) (Figure 2B). We analyzed the haplotype composition at each local site, focusing on the high‐frequency haplotypes H_2_ and H_3_. The results showed that H_3_ had a high frequency in the northwestern part of the Korean Peninsula, while H_2_ had a high frequency in the southeastern part (Figure 2A). At Site 8, the frequencies of the other haplotypes were higher than those of H_2_ and H_3_, indicating a mixed haplotype composition of the northeastern and southeastern populations (Figure 2A).

In the pairwise *F*_ST_ among local sites based on MT multi‐gene sequences, the genetic distance ranged from −0.01328 to 0.93501. The analysis based on the genetic distances proposed that the local populations were clustered into two groups: the northwestern group (seven local populations: Sites 2, 7, 8, 9, 10, 11, and 12) and the southeastern group (five local populations: Sites 1, 3, 4, 5, and 6), similar to the results of the median‐joining network (Table 5, upper diagonal). Site 8, which was difficult to group based on the haplotype composition of each local site, was clustered into the northwestern group. In the AMOVA results, genetic variation among groups (the northwestern group and the southeastern group) accounted for 68.88% (*F*_CT_ = 0.68877, *p* = 0.00196). Variation among local sites within groups was relatively low at 3.64% (*F*_SC_ = 0.11646, *p* = 0.00000), while that within local sites was 27.48% (*F*_ST_ = 0.72517, *p* = 0.00000) (Table 6, MT). Specifically, the *F*‐value among groups was relatively high, seemingly reflecting the two high‐frequency haplotypes connected by eight median vectors in the median‐joining network.

**TABLE 5:** Pairwise *F*_ST_ distances of regional populations of *Ophraella communa* that have invaded Korea.

Site No.	1	2	3	4	5	6	7	8	9	10	11	12	GDML
1	—	0.65343	0.17795	0.04164	0.03961	−0.00646	0.67892	0.45774	0.67051	0.60425	0.68593	0.42942	0.701282 ± 0.387569
2	0.02245	—	0.92649	0.82988	0.47152	0.69823	0.00703	0.25054	0.00000	0.02281	0.00000	0.12812	0.623993 ± 0.349810
3	0.02874	0.03150	—	0.02810	0.38366	0.09237	0.93027	0.79090	0.93102	0.91147	0.93501	0.76544	0.630037 ± 0.352766
4	0.03272	0.01779	0.04367	—	0.21344	−0.00152	0.84366	0.65811	0.84036	0.79763	0.84961	0.63368	0.689011 ± 0.381579
5	0.04548	0.04243	0.02422	0.04665	—	0.12067	0.50177	0.26395	0.49180	0.41473	0.51044	0.22108	0.692491 ± 0.383278
6	0.02840	0.03449	0.03334	0.01186	0.04012	—	0.71559	0.54436	0.70922	0.66546	0.72070	0.51892	0.681685 ± 0.378002
7	0.04555	0.03185	0.08267	0.04253	0.06264	0.02738	—	0.27017	−0.00323	−0.01328	0.00000	0.13896	0.658974 ± 0.366910
8	0.02614	0.00487	0.03439	0.00423	0.02645	0.00162	0.02013	—	0.26946	0.19587	0.28716	0.06095	0.689744 ± 0.389398
9	0.04761	0.03958	0.07311	0.03100	0.07456	0.02073	0.04160	0.01483	—	0.03116	0.00000	0.14001	0.648901 ± 0.361987
10	0.07351	0.05219	0.13841	0.09116	0.13841	0.09641	0.03664	0.07085	0.06554	—	0.03886	0.09202	0.522344 ± 0.300012
11	0.05657	0.03743	0.11111	0.05969	0.11329	0.06801	0.03209	0.04693	0.04856	0.00028	—	0.15118	0.572344 ± 0.324531
12	0.06470	0.05271	0.12117	0.05805	0.12885	0.06486	0.04729	0.04462	0.02751	0.00877	0.00963	—	0.564530 ± 0.327963

Abbreviations: GDML, gene diversity based on microsatellite loci; lower side, microsatellite loci; upper side, mitochondrial genes.

**TABLE 6:** AMOVA of mitochondrial genes and microsatellites of *Ophraella communa* that have invaded Korea.

Source of variation	Sum of squares	Variance components	Percentage of variation	Fixation indices	*p*
MT					
Among groups	3116.934	14.05814Va	68.88	*F*_CT_ = 0.68877	0.00196
Among local sites within group	324.729	0.74298Vb	3.64	*F*_SC_ = 0.11696	0.00000
Within local sites	2400.834	5.60943Vc	27.48	*F*_ST_ = 0.72517	0.00000
MS					
Among groups	36.999	0.10375	4.30647	*F*_CT_ = 0.04306	0.00000
Among local sites within group	39.119	0.05646	2.34355	*F*_SC_ = 0.02449	0.00000
Among individuals within local sites	476.707	−0.14751	−6.12266	*F*_IS_ = −0.06559	1.00000
Within individuals	571.500	2.39649	99.47264	*F*_IT_ = 0.00527	0.87195

Abbreviations: MS, microsatellite loci genotype data; MT, mitochondrial DNA sequences data.

**FIGURE 3:**
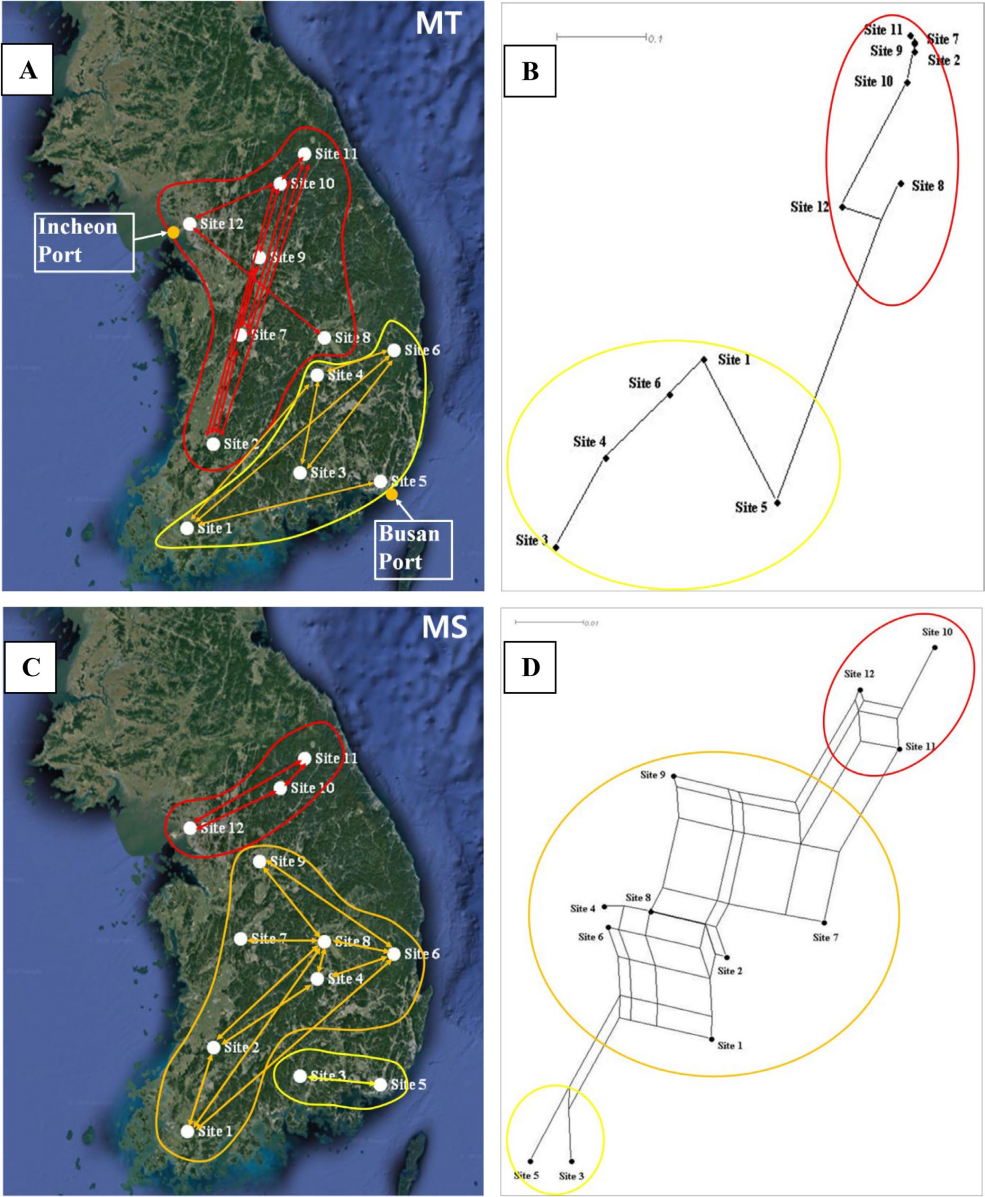
Genetic structure of regional populations based on pairwise *F*_ST_. (A) Genetic relationship map based on mitochondrial genes; (B) Neighbor‐net network based on mitochondrial genes; (C) Genetic relationship map of local populations based on microsatellite loci; (D), Neighbor‐net network based on microsatellite loci (Satellite map ver. 2015 provided by National Geographic Information Institute under Ministry of Land, Infrastructure and Transport of Korea).

A detailed comparison of pairwise *F*_ST_ genetic distances revealed that Sites 2, 7, 9, 10, and 11 in the northwestern group exhibited smaller genetic distances than those between Sites 8 and 12. Similarly, Sites 1, 4, and 6 in the southeastern group had smaller genetic distances than those between Sites 3 and 5. These results were also clearly shown in the relationship map among local populations and the neighbor‐net network constructed based on the pairwise *F*_ST_ (Figure 3A,B). In the neighbor‐net network, five genetically close local populations (Sites 2, 7, 9, 10, and 11) in the northwestern group with a relatively low genetic distance were closely clustered on one side, and three genetically close local populations (Sites 1, 4, and 6) in the southeastern group were linearly connected between Sites 3 and 5 (Figure 3B).

### 3.2 Population Structure Using Multi‐Locus Genotype Data

#### 3.2.1 NGS Sequencing and Microsatellite Loci Screening

NGS was performed to obtain the reference whole genome sequence of the ragweed leaf beetle required for microsatellite loci screening. Using the MGISE‐Q2000 platform, 37,990,794,126 bp from 127,013,948 reads (average length 150 bp) were obtained from 20 ragweed leaf beetle individuals and assembled into 5,917,452 scaffolds (average length 249 bp, longest scaffold 109,890 bp, shortest scaffold 100 bp) (Table S1). The obtained scaffolds contained 80,753 microsatellite loci. Among them, 61,025 were confirmed to be di‐, tri‐, or tetranucleotide repeat loci (Table S1). Seventeen microsatellite loci were screened using the screening criteria reported in the materials and methods. Following PCR testing, markers for nine loci were finally selected, including microsatellite loci nos. 101,695, 9248, 2,689,147, 1,571,517, 558,867, 580,013, 14,631, 11,007, and 420,873 (Table 3).

#### 3.2.2 Microsatellite Marker Assessment and Genetic Characteristics

We tested for null alleles and PCR errors using the nine microsatellite markers selected for the ragweed leaf beetle individuals collected from the 12 local sites. We confirmed the presence of null alleles in three markers: 14631, 420,873, and 558,867. The null allele frequencies for 14,631, 420,873, and 558,867 were up to 0.3493, 0.1496, and 0.3409, respectively (Table S2). Microsatellite analysis revealed that the null allele frequencies were almost always *p* < 0.40 but usually *p* < 0.20 (Dakin and Avise 2004). When microsatellite null alleles are uncommon to rare (*p* < 0.20), their presence causes a slight underestimation of the average exclusion probability at a locus. However, this is usually of insufficient magnitude to warrant great concern. However, for *p* > 0.20, the mean “estimated with null” exclusion probability can be much higher than the “true” and “estimated without null” values (Dakin and Avise 2004). Therefore, two markers, 14,631 and 558,867, with *p* > 0.20, were excluded from genetic diversity analysis in this study. Finally, we estimated genetic diversity indexes, including gene diversity, number of genotypes and alleles, observed and expected heterozygosity, exact *p*‐value, squared deviation, and Garza–Williamson index, using seven markers: 9248, 11,007, 101,695, 420,873, 580,013, 1,571,517, and 2,689,147.

Gene diversity values in invasive ragweed leaf beetle populations in the Korean Peninsula ranged from 0.522344 ± 0.300012 (Site 11) to 0.71282 ± 0.387569 (Site 1) (Table 5). The observed heterozygosity ranged from 0.20000 (Site 3 of marker 101,695) to 1.00000 (Sites 3, 4, 5, 7, and 8 of marker 580,013; Site 7 of marker 689,147) and showed relatively high values for markers 580,013 and 689,147 (Table S3). Expected heterozygosity ranged from 0.27821 (Site 10 of marker 9248) to 0.83974 (Site 9 of marker 420,873) (Table S3). The genetic diversity of each marker was relatively low, ranging from 3 to 10 alleles (Table S3). The exact *p*‐values of HWE were calculated for each local population after Bonferroni correction (*p* = 0.000595). Deviation from HWE was not detected in the exact *p*‐values, except for marker 580,013 at Site 7 (*p* = 0.00044). In addition, six of the seven markers used for analyzing the genetic diversity of invasive ragweed beetle populations showed relatively low exact *p*‐values of *p* < 0.1 [Table S3; 9248 (Sites 3 and 12), 101,695 (Sites 3 and 4), 420,873 (Sites 5, 8, and 9), 580,013 (Sites 1, 3, 5, 7, 8, and 9), 1,571,517 (Site 7), and 689,147 (Site 12)]. Similar to this result, observed heterozygosity values lower than expected suggest significant homozygosity, which implies the presence of null alleles or allelic dropout, allele linkage, or inbreeding (Damm et al. 2015; Kang et al. 2017). If the violation were a consequence of inbreeding, we would have expected to observe such a phenotype at several or all loci and not just at a single locus (Damm et al. 2015; Kang et al. 2017). However, in our results, it was difficult to consider inbreeding or sib sampling within groups because some markers with low exact *p*‐values showed lower expected than observed heterozygosity values. Thus, we examined the genetic bottleneck of each microsatellite locus using the Garza–Williamson index, calculated using Arlequin ver. 3.1 (Garza and Williamson 2001; Excoffier et al. 2005). The whole local population analyzed in this study showed an M‐ratio less than 0.7 in all markers used, and we might infer that ragweed leaf beetles invading the Korean Peninsula are undergoing a genetic bottleneck. In the exact *p*‐value analysis, although marker 580,013 at Site 7 deviated from HWE, we consider that our case occurred because the invasive populations exhibited the same characteristics as the pioneer population (Dlugosch and Parker 2008). Therefore, we suggest that the seven markers developed in this study may be useful for analyzing the genetic structure of invasive ragweed leaf beetles.

#### 3.2.3 Pairwise *F*_ST_ Genetic Distances

Pairwise *F*_ST_ among local sites based on MS multi‐locus genotype data revealed that the genetic distance ranged from −0.00028 to 0.13841. The local populations were clustered into three groups through the analysis based on the genetic distances (Table 5, lower diagonal): northern (three local populations: Sites 10, 11, and 12), middle‐western (seven local populations: Sites 1, 2, 4, 6, 7, 8, and 9), and southern (two local populations: Sites 3 and 5) groups, similar to the neighbor‐net network results (Figure 3D). The AMOVA results showed that the percentage of variation among groups (northern, middle‐western, and southern groups) was 4.3064% (*F*_CT_ = 0.04306, *p* = 0.00000), which was higher than that among local sites within groups (2.34355%; *F*_SC_ = 0.02449, *p* = 0.00000), showing a clear difference among groups (Table 6, MS). The *F*_IS_ value was negative (−0.0.06559, *p* = 1.00000), indicating that the inbreeding rate within the local population was low (Table 6, MS). The relationship map of local populations and the neighbor‐net network constructed based on pairwise *F*_ST_ demonstrated that although the northern group exhibited low genetic distances overall, Sites 10 and 12 were relatively closer to each other than to Site 11. The southern group was distinguished from the other local populations based on genetic distance. Sites 4, 6, and 8 in the middle‐western group were closer than those of the other local populations (Sites 1, 2, 9, and 7) in terms of genetic distance (Figure 3C,D).

#### 3.2.4 Bayesian Clustering

**FIGURE 4:**
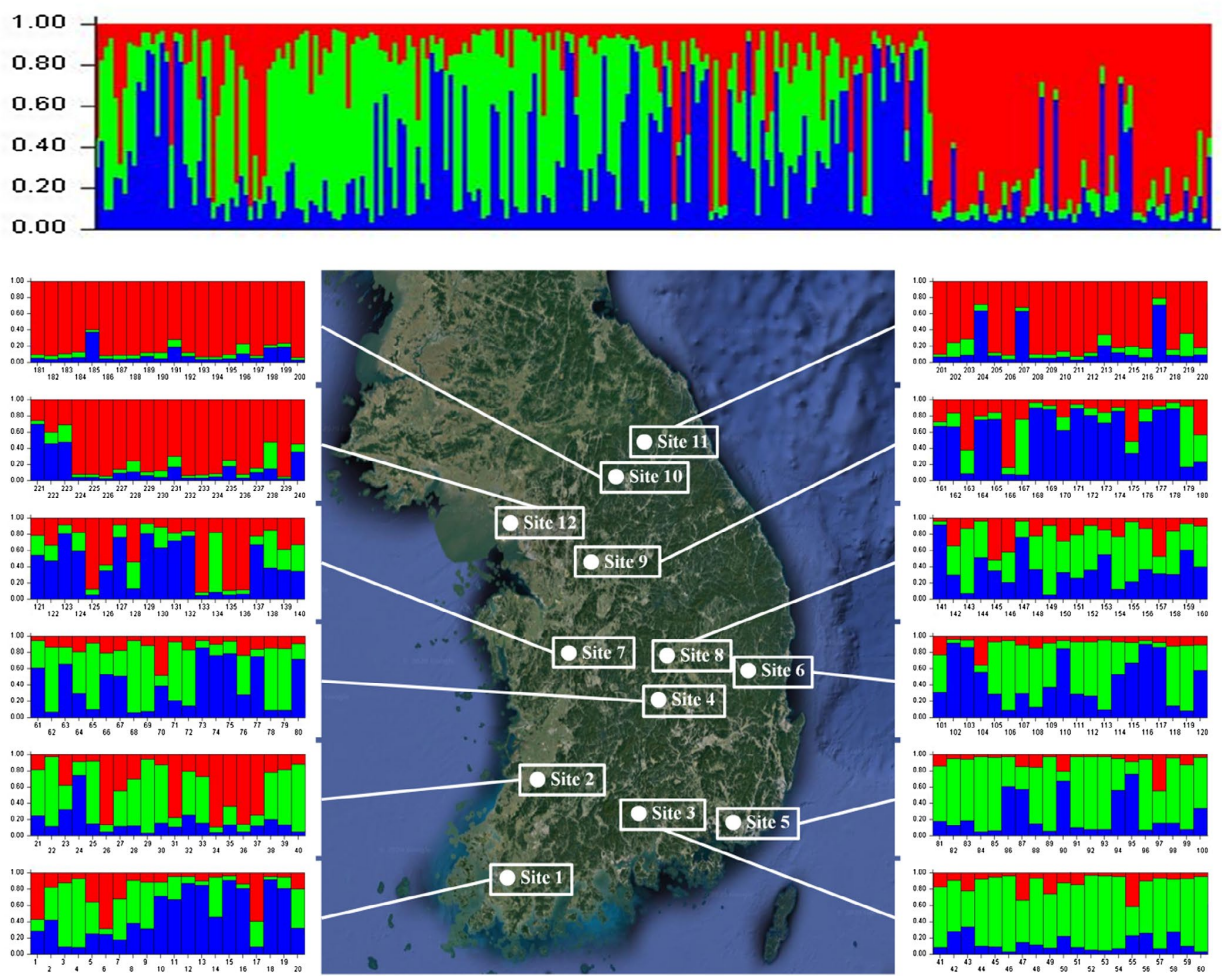
Bar plots estimated using STRUCTURE for 240 individuals from 12 sites. The optimal *K* was estimated as 3 based on the ad hoc statistic Δ(*K*). The individual bar plots for each local population showed high frequencies of component 1 in Sites 10, 11, and 12, component 2 in Sites 3 and 5, and mixed component in Sites 1, 2, 4, 6, 7, 8, and 9 (Satellite map ver. 2015 provided by National Geographic Information Institute under Ministry of Land, Infrastructure and Transport of Korea).

In the model‐based Bayesian analysis, *K* was estimated by varying it from 1 to 8. The ad hoc statistic Δ(*K*) (Evanno et al. 2005) indicated the maximum level of structure in the three components (represented by red, green, and blue), consistent with the results based on pairwise *F*_ST_ (Figure S1). *F*_ST_ of each component is 0.2048 in component 1 (represented by red), 0.1061 in component 2 (represented by green), and 0.0068 in component 3 (represented by blue), respectively. Thus, we confirmed that at least three invasive ragweed leaf beetle populations were present in the Korean Peninsula based on MS multi‐locus genotype data. Comparison of the individual bar plots for each local population showed high frequencies of component 1 (represented by red) in the northern group (Sites 10, 11, and 12), component 2 (represented by green) in the southern group (Sites 3 and 5), and mixed component in the middle‐western group (Sites 1, 2, 4, 6, 7, 8, and 9) (Figure 4). The northern and southern groups were clearly distinguished as component 1 and 2, while the middle‐western group showed mixed component. Specifically, in the local populations of the middle‐western group, three populations (Sites 4, 6, and 8) showed mixed patterns with component represented by blue and green, while Site 2 displayed mixed patterns with component by green and red. Two populations (Sites 7 and 9) showed slightly mixed patterns with component by blue and red. Site 1 exhibited component by blue, green, and red (Figure 4). These findings are consistent with those of the neighbor‐net network (Figure 3D). Analysis using MS multi‐locus genotype data indicated that three local populations (Sites 4, 6, and 8) might be mixed with populations from the southern group (Sites 3 and 5), two local populations (Sites 7 and 9) with populations from the northern group (Sites 10, 11, and 12), Site 2 with northern and southern groups, and Site 1 with all groups.

## 4 Discussion

### 4.1 Comparison Between Mitochondrial Sequence and Microsatellite Loci Data Results

The MT and MS data did not completely coincide, indicating the difference in the estimated number of populations. In the MT data, the invasive ragweed leaf beetles in the Korean Peninsula were divided into two groups: northwestern (Sites 2, 7, 8, 9, 10, 11, and 12) and southeastern (Sites 1, 3, 4, 5, and 6) groups (Figure 3A). However, in the MS data, they were divided into three groups: northern (Sites 10, 11, and 12), middle‐western (Sites 1, 2, 4, 6, 7, 8, and 9), and southern (Sites 3 and 5) groups (Figure 3C). The results were cross‐checked using AMOVA, and the groups estimated using MT and MS data‐based analysis were considered valid (Table 6).

In Korea, the ragweed leaf beetle is an exotic species, and some populations from adjacent countries of Korea as well as North America might be introduced into Korea by sea‐transportation (Ohno 1997; Wang and Chiang 1998; Sohn et al. 2002). This situation may be similar to that of a founder event (Dlugosch and Parker 2008). Thus, the discrepancy between the MT and MS data in our study may be attributed to founder events, which may explain the high divergence of peripheral populations (Tollefsrud et al. 2009). The MT data‐based results revealed that the sequence divergence of the sampled individuals was relatively high, with a maximum of 3.3%. In the median‐joining network, the two estimated groups were connected by eight median vectors (unobserved sequences) (Figure 2B). However, in the MS data, the northern and southern groups were clearly distinguished by genetic patterns, whereas the middle‐western group showed a mixed genetic pattern (Figure 4). Two explanations could account for the apparent discordance between the two datasets: insufficient time for nuclear gene lineage sorting and introgression (Zink 2008). The coalescence theory predicts that in cases where groups have been isolated for a sufficient duration, an mtDNA gene tree may achieve reciprocal monophyly, whereas a nuclear gene tree may not (Scribner et al. 2003; Zink 2008). Our results showed the distinct genetic differences between MT data and MS data. Specifically, the individual bar plot of the middle‐western group suggests that individuals from Sites 4, 5, and 8 may be mixed with the southern group; individuals from Sites 7 and 9 with the northern group; individuals from Site 2 with the northern and southern groups; and individuals from Site 1 with the northern, southern and middle‐western groups (Figure 4). Hybridization causes group boundary decay, especially for nuclear loci, because alleles are spread by males and females (Zink 2008). The patterns of mtDNA variation can be influenced by several factors, including the stochastic nature of the coalescence process, selection, and sex‐biased dispersal. Nuclear loci are required to test the patterns of mtDNA differentiation, although it has been established that the longer coalescence times of nuclear loci prohibit them from corroborating mtDNA patterns (Moore 1995; Zink and Barrowclough 2008; Zink 2008). Therefore, we considered that the ragweed leaf beetle invasion pathway might be inferred by focusing on MT data while also considering insights from MS data.

### 4.2 Ragweed Leaf Beetle Spreading Pathways in the Korean Peninsula

The Korean Peninsula, bordered by the sea on three sides, is geopolitically located in East Asia, or the eastern part of the Eurasian continent (Nestor 1977; GSK 1998; Nemeth 2003). Since the Korean War (1950–1953), a demilitarized zone (DMZ) has been established, and entering the Korean Peninsula by land from the north is not possible (Nestor 1977; GSK 1998; Nemeth 2003). Thus, most international trade is conducted in the form of maritime trade using international vessels (99.7%) or air trade using international airplanes (0.3%) (MOLIT 2016). Therefore, most invasive insect pests have been introduced to the Korean Peninsula by hitchhiking on vessels (Kang et al. 2020, 2023, 2024). In addition, the geographical features of the Korean Peninsula are characterized by a primarily mountainous east and a west comprising low plains (Nestor 1977; GSK 1998; S. J. Park 2014). Major highways are connected to industrialization hubs, such as major national industrial complexes (52 locations) and inland logistics bases (seven locations) in each region (ILIS 2016; NLIC 2025). This geographical situation has recently enabled the invasion and spread of hitchhiking insect pests into the Korean Peninsula, including *Melanoplus differentialis* (Acrididae, Orthoptera), *Pochazia shantungensis* (Ricaniidae, Hemiptera), *Metcalfa pruinosa* (Flatidae, Hemiptera), *Lycorma delicatula* (Fulgoridae, Hemiptera), *Leptoglossus occidentalis* (Coeridae, Hemiptera), and *Vespa velutina* (Vespidae, Hymenoptera) (Han et al. 2008; Lee and Wilson 2010; Choi et al. 2012; Yoon et al. 2012; Kang et al. 2022). Ragweed leaf beetles are believed to have been introduced through this pathway; however, the exact pathway of invasion and spread is yet to be confirmed (Sohn et al. 2002).

Generally, the introduction and spread of invasive herbivorous pests mostly occur along with their host plants. However, in the case of ragweed beetles, ragweed, the host plant, was reportedly introduced during and around the Korean War (Lee 1968; S. H. Park 1995; Kim et al. 2017), and the ragweed leaf beetle was discovered on the Korean Peninsula and is believed to have spread nationwide through multiple invasions from 2000 with the increase in international trade (Sohn et al. 2002; UNtd 2025). Therefore, based on the results of the present and previous studies (Lee 1968; S. H. Park 1995; Sohn et al. 2002; Kim et al. 2017), we inferred the invasive pathway of the ragweed leaf beetle into domestic areas within the Korean Peninsula.

Based on the results of the present study, the exotic ragweed leaf beetle in Korea is divided into two groups: northwestern and southeastern (Figures 2 and 3A). Incheon Port and Busan Port might be the entry points for each group into the Korean Peninsula (Figure 3A) (Sohn et al. 2002), and each invasive population might spread to each local site through transport, such as vehicles, tractor‐trailers, or trains. Through flight activity tests, the adult ragweed leaf beetle can reportedly travel 21.4 km for males and 25.4 km for females daily, traveling an average of 77 km annually (Yamamura et al. 2007; Tanaka and Yamanaka 2009) and is strongly attracted to the host plant ragweed (Lee et al. 2023). Due to these ecological habits, ragweed leaf beetles that have spread inland from entry points may move to local areas where ragweed inhabits. In addition, the distinct grouping of this exotic species might be caused by the geographical features of the Korean Peninsula, which is characterized by a mountainous area extending from the northeast to the southwest (the Taebaek and Nangnim Mountain Ranges, Charyeong Mountain Ranges, Noryeong Mountain Ranges, and Sobaek Mountain Ranges) (GSK 1998; Nemeth 2003; S. J. Park 2014; Kwon et al. 2016). These geographical features can also affect logistics operations. Tractor‐trailers or trains transporting containers might generally deliver goods to the northwestern part of Korea from Incheon Port and to the southeastern part from Busan Port rather than crossing the mountain ranges that extend from the northeast to the southwest. In addition, coastal transport may also be utilized (MOLIT 2016).

In conclusion, based on ecological traits and geographical features, the invasive pathways of ragweed leaf beetles suggest that the species may have entered the Incheon and Busan ports of the Korean Peninsula by hitchhiking on international vessels. They then spread into the domestic regions of the Korean Peninsula via transport systems, such as tractor‐trailers or trains that carry containers from ports, finally dispersing into the local areas of each region through the strong attraction of individuals to the host plants. Therefore, we suggest that the invasive pathways of ragweed leaf beetles have facilitated their spread into the domestic regions of Korea. We expect that our results will aid in identifying key points to prevent the inland spread of exotic species.

## Author Contributions

**Tae Hwa Kang:** conceptualization (equal), data curation (lead), formal analysis (lead), investigation (lead), methodology (lead), project administration (supporting), resources (lead), software (lead), supervision (equal), validation (equal), visualization (equal), writing – original draft (lead), writing – review and editing (lead). **Sun Jae Park:** conceptualization (equal), data curation (supporting), formal analysis (supporting), funding acquisition (lead), investigation (supporting), methodology (supporting), project administration (lead), resources (supporting), writing – original draft (supporting), writing – review and editing (equal).

## Funding

This work was supported by a grant from the National Institute of Biological Resources (NIBR), funded by the Ministry of Environment (MOE), the Korean Government (NIBR202211101).

## Conflicts of Interest

The authors declare no conflicts of interest.

## Supporting information


**Figure S1:** Ad hoc statistics Δ(*K*) and mean LnP(*K*) ± Stdev based on LnP(D) estimated from 20 iterations of each *K* using StructureSelector. The ad hoc statistics exhibited a signal, with *K* = 3 as the optimal value.

**Table S1:** Summary of next‐generation sequencing results and obtained microsatellite loci of *Ophraella communa* that have invaded Korea.

**Table S2:** Results of the null allele test and PCR error analysis in the nine filtered markers.

**Table S3:** Characteristics of the seven polymorphic microsatellite loci developed from *Ophraella communa* that have invaded Korea.

## Data Availability

DNA sequences of COI gene are available in GenBank (OL690593–OL691056); DNA sequences of NADH5 gene are available in GenBank (OL754676–OL755120); DNA sequences of ATP6/ATP8 gene are available in GenBank (OL755121–OL755584).
