# Supplementary material for: Integrative Analysis of the Invasive Pathways of the Ragweed Leaf Beetle Ophraella communa LeSage 1986 (Coleoptera, Chrysomelidae) Into Domestic Areas of the Korean Peninsula
Source: Ecol Evol. 2026 Jun 18;16(6):e73876. doi: 10.1002/ece3.73876 (PMC13277759; doi:10.1002/ece3.73876)
Supplement: Supplementary file 1 — Figure S1: Ad hoc statistics Δ(K) and mean LnP(K) ± Stdev based on LnP(D) estimated from 20 iterations of each K using StructureSelector. The ad hoc statistics exhibited a signal, with K = 3 as the optimal value. [file ECE3-16-e73876-s002.docx]

**Supporting Information**

**Figure S1.** Ad hoc statistics Δ(*K*) and mean LnP(*K*)±Stdev based on LnP(D) estimated from 20 iterations of each *K* using StructureSelector. The ad hoc statistics exhibited a signal, with *K* = 3 as the optimal value.

**B**

**
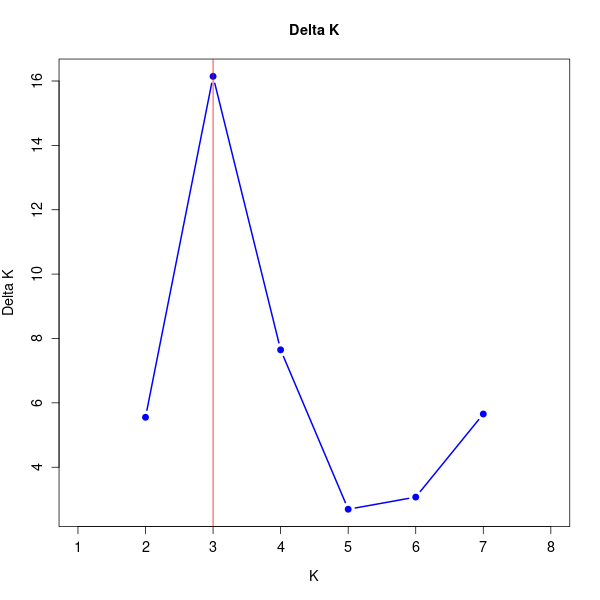

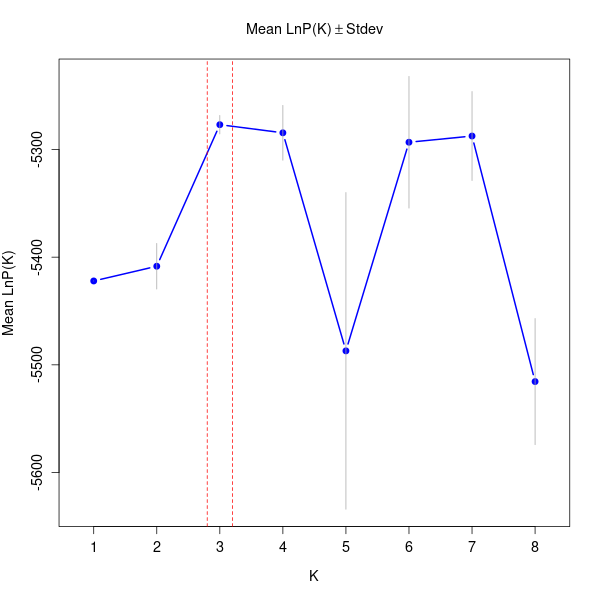
**

**A**
