# Supplementary material for: Integrative Analysis of the Invasive Pathways of the Ragweed Leaf Beetle Ophraella communa LeSage 1986 (Coleoptera, Chrysomelidae) Into Domestic Areas of the Korean Peninsula
Source: Ecol Evol. 2026 Jun 18;16(6):e73876. doi: 10.1002/ece3.73876 (PMC13277759; doi:10.1002/ece3.73876)
Supplement: Supplementary file 2 — Table S1: Summary of next‐generation sequencing results and obtained microsatellite loci of Ophraella communa that have invaded Korea. [file ECE3-16-e73876-s001.docx]

**Supporting Information**

**Table S1.** Summary of next-generation sequencing results and obtained microsatellite loci of *Ophraella communa* that have invaded Korea.

| **Sequencing data summary** | | | **Assembled genome summary** | | | |
| --- | --- | --- | --- | --- | --- | --- |
| **RN** | **RAL** | **Total bp** | | **SN** | **SAL** | **Scaffolds bp** |
| 127,013,948 | 150 | 37,990,794,126 | | 5,917,452 | 249 | 1,478,492,318 |
| **RNN** | | | **PMSN** | | | |
| Di-nucleotides | | | 25,621 | | | |
| Tri-nucleotides | | | 27,683 | | | |
| Tetra-nucleotides | | | 7,721 | | | |
| Penta-nucleotides | | | 16,565 | | | |
| Hexa-nucleotides | | | 1,126 | | | |
| Hepta-nucleotides | | | 1,536 | | | |
| Octa-nucleotides | | | 501 | | | |
| **TPMSN** | | | **80,753** | | | |

RN, Number of reads; RAL, Read average length; SN, Number of scaffolds; SAL, Scaffolds average length; RNN, Number of repeated nucleotide; PMSN, Number of perfect microsatellite sequences.
