# Supplementary material for: Integrative Analysis of the Invasive Pathways of the Ragweed Leaf Beetle Ophraella communa LeSage 1986 (Coleoptera, Chrysomelidae) Into Domestic Areas of the Korean Peninsula
Source: Ecol Evol. 2026 Jun 18;16(6):e73876. doi: 10.1002/ece3.73876 (PMC13277759; doi:10.1002/ece3.73876)
Supplement: Supplementary file 3 — Table S2: Results of the null allele test and PCR error analysis in the nine filtered markers. [file ECE3-16-e73876-s004.docx]

**Supporting Information**

**Table S2.** Results of the null allele test and PCR error analysis in the nine filtered markers.

| **ML** | **Site 1** |  | **Site 2** |  | **Site 3** |  | **Site 4** |  |
| --- | --- | --- | --- | --- | --- | --- | --- | --- |
|  | **NP** | **Freq** | **NP** | **Freq** | **NP** | **Freq** | **NP** | **Freq** |
| 9248 | no | 0.0528 | no | 0.0372 | no | -0.0977 | no | -0.0946 |
| 11007 | no | -0.0770 | no | -0.3787 | no | -0.1955 | no | 0.0018 |
| 14631^*^ | yes | 0.3493 | yes | 0.3264 | yes | 0.2220 | no | 0.1095 |
| 101695 | no | 0.0728 | no | 0.0332 | no | 0.1600 | no | 0.1211 |
| 420873 | no | 0.0256 | no | 0.0205 | no | 0.0263 | no | 0.0209 |
| 558867 | no | 0.1514 | no | 0.0896 | no | 0.0578 | no | 0.0298 |
| 580013 | no | -0.2196 | no | -0.2711 | no | -0.3524 | no | -0.3365 |
| 1571517 | no | 0.0516 | no | 0.0465 | no | -0.0048 | no | 0.0842 |
| 2689147 | no | 0.0460 | no | 0.0281 | no | -0.1238 | no | -0.1320 |
| **ML** | **Site 5** |  | **Site 6** |  | **Site 7** |  | **Site 8** |  |
|  | **NP** | **Freq** | **NP** | **Freq** | **NP** | **Freq** | **NP** | **Freq** |
| 9248 | no | -0.0966 | no | 0.0019 | no | -0.0520 | no | -0.0625 |
| 11007 | no | -0.1188 | no | -0.0172 | no | 0.0110 | no | -0.1421 |
| 14631^*^ | no | 0.1032 | yes | 0.2587 | yes | 0.2340 | yes | 0.1617 |
| 101695 | no | -0.0067 | no | 0.0988 | no | -0.1559 | no | 0.0719 |
| 420873 | no | 0.1362 | no | -0.0262 | no | -0.1654 | yes | 0.1496 |
| 558867^*^ | no | 0.0581 | yes | 0.3409 | no | 0.0655 | yes | 0.2746 |
| 580013 | no | -0.3437 | no | -0.3244 | no | -0.4565 | no | -0.3511 |
| 1571517 | no | -0.0241 | no | 0.0575 | no | -0.1610 | yes | 0.1585 |
| 2689147 | no | -0.0220 | no | -0.0764 | no | -0.1688 | no | -0.0716 |
| **ML** | **Site 9** |  | **Site 10** |  | **Site 11** |  | **Site 12** |  |
|  | **NP** | **Freq** | **NP** | **Freq** | **NP** | **Freq** | **NP** | **Freq** |
| 9248 | no | -0.0117 | no | -0.1533 | no | -0.0383 | no | 0.1347 |
| 11007 | no | 0.0691 | no | 0.0305 | no | -0.3548 | no | -0.0384 |
| 14631^*^ | yes | 0.1987 | yes | 0.2207 | yes | 0.3062 | no | 0.1753 |
| 101695 | no | -0.0471 | no | -0.0822 | no | 0.0792 | no | 0.0386 |
| 420873 | no | -0.0546 | no | -0.1141 | no | 0.0271 | no | -0.0192 |
| 558867 | no | 0.1455 | yes | -0.0766 | no | -0.1020 | no | -0.1633 |
| 580013 | no | -0.4311 | no | -0.4329 | no | -0.5026 | no | -0.5164 |
| 1571517 | no | -0.1887 | no | 0.0331 | no | 0.0142 | no | 0.0072 |
| 2689147 | no | -0.0724 | no | -0.1199 | no | -0.1539 | no | 0.0451 |

ML, microsatellite loci; NP, null present; the asterisk (*), marker with null allele
