# Supplementary material for: Integrative Analysis of the Invasive Pathways of the Ragweed Leaf Beetle Ophraella communa LeSage 1986 (Coleoptera, Chrysomelidae) Into Domestic Areas of the Korean Peninsula
Source: Ecol Evol. 2026 Jun 18;16(6):e73876. doi: 10.1002/ece3.73876 (PMC13277759; doi:10.1002/ece3.73876)
Supplement: Supplementary file 4 — Table S3: Characteristics of the seven polymorphic microsatellite loci developed from Ophraella communa that have invaded Korea. [file ECE3-16-e73876-s003.docx]

**Supporting Information**

**Table S3.** Characteristics of the seven polymorphic microsatellite loci developed from *Ophraella communa* that have invaded Korea.

| **ML** | **SN** | **GN** | **AN** | **H_Obs_** | **H_Exp_** | **GWI** | ***P*-value** | **S.D.** | **ML** | **SN** | **GN** | **AN** | **H_Obs_** | **H_Exp_** | **GWI** | ***P*-value** | **S.D.** |
| --- | --- | --- | --- | --- | --- | --- | --- | --- | --- | --- | --- | --- | --- | --- | --- | --- | --- |
| **9248** | 1 | 20 | 10 | 0.70000 | 0.82179 | 0.09174 | 0.03779 | 0.00025 | **11007** | 1 | 20 | 7 | 0.75000 | 0.68974 | 0.11111 | 0.53241 | 0.00041 |
|  | 2 | 20 | 5 | 0.55000 | 0.63333 | 0.06494 | 0.17017 | 0.00043 |  | 2 | 20 | 6 | 0.70000 | 0.54487 | 0.12766 | 0.80072 | 0.00047 |
|  | 3 | 20 | 4 | 0.65000 | 0.54487 | 0.03670 | 0.05524 | 0.00023 |  | 3 | 20 | 4 | 0.75000 | 0.58333 | 0.08889 | 0.45330 | 0.00045 |
|  | 4 | 20 | 7 | 0.85000 | 0.74872 | 0.07527 | 0.96082 | 0.00020 |  | 4 | 20 | 7 | 0.65000 | 0.67564 | 0.13208 | 0.32055 | 0.00041 |
|  | 5 | 20 | 5 | 0.70000 | 0.63718 | 0.04132 | 0.36711 | 0.00051 |  | 5 | 20 | 7 | 0.85000 | 0.73846 | 0.14894 | 0.41901 | 0.00049 |
|  | 6 | 20 | 5 | 0.70000 | 0.75641 | 0.06849 | 0.15097 | 0.00038 |  | 6 | 20 | 7 | 0.80000 | 0.78974 | 0.14894 | 0.56486 | 0.00051 |
|  | 7 | 20 | 6 | 0.60000 | 0.61154 | 0.04651 | 0.66825 | 0.00039 |  | 7 | 20 | 8 | 0.65000 | 0.68077 | 0.17778 | 0.72880 | 0.00046 |
|  | 8 | 20 | 8 | 0.75000 | 0.73846 | 0.10390 | 0.58739 | 0.00049 |  | 8 | 20 | 5 | 0.80000 | 0.68974 | 0.10638 | 0.36804 | 0.00052 |
|  | 9 | 20 | 6 | 0.65000 | 0.65513 | 0.07407 | 0.54683 | 0.00048 |  | 9 | 20 | 5 | 0.60000 | 0.70641 | 0.11111 | 0.44464 | 0.00053 |
|  | 10 | 20 | 5 | 0.30000 | 0.27821 | 0.06849 | 1.00000 | 0.00000 |  | 10 | 19 | 6 | 0.52632 | 0.56472 | 0.13333 | 0.10174 | 0.00026 |
|  | 11 | 20 | 5 | 0.45000 | 0.46282 | 0.06494 | 0.23925 | 0.00049 |  | 11 | 20 | 4 | 0.65000 | 0.50897 | 0.08889 | 0.61048 | 0.00047 |
|  | 12 | 20 | 5 | 0.25000 | 0.38974 | 0.04132 | 0.06796 | 0.00022 |  | 12 | 18 | 3 | 0.61111 | 0.60794 | 0.06667 | 0.16510 | 0.00037 |
| **101695** | 1 | 20 | 5 | 0.45000 | 0.52564 | 0.04854 | 0.38209 | 0.00038 | **420873** | 1 | 20 | 7 | 0.70000 | 0.74744 | 0.17073 | 0.32349 | 0.00036 |
|  | 2 | 20 | 5 | 0.50000 | 0.53333 | 0.06098 | 0.16091 | 0.00042 |  | 2 | 20 | 5 | 0.60000 | 0.65256 | 0.33333 | 0.68072 | 0.00055 |
|  | 3 | 20 | 4 | 0.20000 | 0.31026 | 0.03883 | 0.08864 | 0.00028 |  | 3 | 20 | 7 | 0.75000 | 0.80769 | 0.30435 | 0.79707 | 0.00045 |
|  | 4 | 20 | 5 | 0.50000 | 0.68205 | 0.06098 | 0.06539 | 0.00025 |  | 4 | 20 | 8 | 0.70000 | 0.76667 | 0.34783 | 0.38246 | 0.00040 |
|  | 5 | 20 | 8 | 0.60000 | 0.61410 | 0.07767 | 0.23993 | 0.00032 |  | 5 | 19 | 5 | 0.47368 | 0.64438 | 0.23810 | 0.01366 | 0.00012 |
|  | 6 | 20 | 5 | 0.45000 | 0.56410 | 0.04854 | 0.21237 | 0.00036 |  | 6 | 20 | 9 | 0.85000 | 0.83718 | 0.20930 | 0.76326 | 0.00042 |
|  | 7 | 20 | 5 | 0.80000 | 0.64103 | 0.04854 | 0.57497 | 0.00045 |  | 7 | 19 | 5 | 0.84211 | 0.67141 | 0.12821 | 0.29449 | 0.00053 |
|  | 8 | 20 | 6 | 0.50000 | 0.57179 | 0.05825 | 0.11164 | 0.00029 |  | 8 | 20 | 9 | 0.55000 | 0.80769 | 0.39130 | 0.00439 | 0.00007 |
|  | 9 | 20 | 6 | 0.75000 | 0.70513 | 0.07317 | 0.72606 | 0.00043 |  | 9 | 20 | 9 | 0.90000 | 0.83974 | 0.47368 | 0.07047 | 0.00027 |
|  | 10 | 20 | 3 | 0.60000 | 0.53718 | 0.03659 | 1.00000 | 0.00000 |  | 10 | 20 | 3 | 0.75000 | 0.62949 | 0.27273 | 0.79623 | 0.00038 |
|  | 11 | 20 | 5 | 0.50000 | 0.58846 | 0.06098 | 0.38445 | 0.00052 |  | 11 | 20 | 6 | 0.60000 | 0.64103 | 0.40000 | 0.91406 | 0.00026 |
|  | 12 | 20 | 4 | 0.60000 | 0.65000 | 0.04878 | 0.35244 | 0.00042 |  | 12 | 20 | 8 | 0.75000 | 0.76026 | 0.42105 | 0.15302 | 0.00029 |
| **580013** | 1 | 20 | 7 | 0.95000 | 0.74359 | 0.17949 | 0.02069 | 0.00012 | **1571517** | 1 | 20 | 5 | 0.55000 | 0.63846 | 0.16129 | 0.12686 | 0.00033 |
|  | 2 | 20 | 8 | 0.95000 | 0.70128 | 0.53333 | 0.34158 | 0.00039 |  | 2 | 20 | 5 | 0.55000 | 0.60256 | 0.16129 | 0.37783 | 0.00046 |
|  | 3 | 20 | 5 | 1.00000 | 0.71026 | 0.45455 | 0.01730 | 0.00011 |  | 3 | 20 | 6 | 0.65000 | 0.66410 | 0.19355 | 0.45190 | 0.00066 |
|  | 4 | 20 | 9 | 1.00000 | 0.73462 | 0.19149 | 0.42459 | 0.00050 |  | 4 | 20 | 5 | 0.35000 | 0.43077 | 0.16129 | 0.25029 | 0.00041 |
|  | 5 | 20 | 8 | 1.00000 | 0.72308 | 0.17021 | 0.09878 | 0.00022 |  | 5 | 20 | 8 | 0.80000 | 0.78590 | 0.25806 | 0.10342 | 0.00027 |
|  | 6 | 19 | 7 | 0.89474 | 0.64438 | 0.13208 | 0.14239 | 0.00035 |  | 6 | 20 | 5 | 0.40000 | 0.43205 | 0.16129 | 0.34660 | 0.00048 |
|  | 7 | 20 | 6 | 1.00000 | 0.62564 | 0.54545 | 0.00044 | 0.00002 |  | 7 | 20 | 4 | 0.80000 | 0.66026 | 0.30769 | 0.02053 | 0.00012 |
|  | 8 | 18 | 7 | 1.00000 | 0.65079 | 0.53846 | 0.00636 | 0.00007 |  | 8 | 20 | 6 | 0.40000 | 0.60513 | 0.26087 | 0.11494 | 0.00020 |
|  | 9 | 20 | 5 | 0.95000 | 0.60513 | 0.09804 | 0.00087 | 0.00003 |  | 9 | 20 | 3 | 0.35000 | 0.30385 | 0.23077 | 1.00000 | 0.00000 |
|  | 10 | 20 | 6 | 0.80000 | 0.60128 | 0.54545 | 0.66729 | 0.00058 |  | 10 | 20 | 4 | 0.55000 | 0.62179 | 0.30769 | 0.12854 | 0.00028 |
|  | 11 | 20 | 8 | 0.95000 | 0.69359 | 0.47059 | 0.56324 | 0.00044 |  | 11 | 20 | 4 | 0.45000 | 0.46795 | 0.30769 | 0.40236 | 0.00042 |
|  | 12 | 19 | 6 | 0.94737 | 0.66856 | 0.40000 | 0.12772 | 0.00027 |  | 12 | 20 | 4 | 0.45000 | 0.45769 | 0.30769 | 0.61041 | 0.00049 |
| **689147** | 1 | 20 | 5 | 0.65000 | 0.74231 | 0.16129 | 0.50335 | 0.00047 |  |  |  |  |  |  |  |  |  |
|  | 2 | 20 | 5 | 0.65000 | 0.70000 | 0.16129 | 0.97178 | 0.00015 |  |  |  |  |  |  |  |  |  |
|  | 3 | 20 | 5 | 0.95000 | 0.78974 | 0.16129 | 0.77825 | 0.00040 |  |  |  |  |  |  |  |  |  |
|  | 4 | 20 | 8 | 0.95000 | 0.78462 | 0.24242 | 0.13771 | 0.00031 |  |  |  |  |  |  |  |  |  |
|  | 5 | 20 | 5 | 0.80000 | 0.76795 | 0.16129 | 0.53890 | 0.00047 |  |  |  |  |  |  |  |  |  |
|  | 6 | 20 | 6 | 0.90000 | 0.81154 | 0.18182 | 0.37778 | 0.00047 |  |  |  |  |  |  |  |  |  |
|  | 7 | 20 | 5 | 1.00000 | 0.78846 | 0.16129 | 0.15211 | 0.00035 |  |  |  |  |  |  |  |  |  |
|  | 8 | 20 | 6 | 0.80000 | 0.72564 | 0.18182 | 0.44954 | 0.00046 |  |  |  |  |  |  |  |  |  |
|  | 9 | 20 | 5 | 0.80000 | 0.72692 | 0.15152 | 0.56889 | 0.00049 |  |  |  |  |  |  |  |  |  |
|  | 10 | 20 | 4 | 0.55000 | 0.47949 | 0.13793 | 1.00000 | 0.00000 |  |  |  |  |  |  |  |  |  |
|  | 11 | 20 | 4 | 0.80000 | 0.64359 | 0.13793 | 0.66324 | 0.00048 |  |  |  |  |  |  |  |  |  |
|  | 12 | 20 | 5 | 0.50000 | 0.52692 | 0.16129 | 0.04277 | 0.00016 |  |  |  |  |  |  |  |  |  |

ML, Microsatellite Loci; SN, Site No.; GN, Number of Genotype; AN, Number of Allele; H_Obs_, Observed Heterozygosity; H_Exp_, Expected Heterozygosity; GWI, Garza-Williamson index; SD, squared deviation; *P*=0.000595 (After Bonferroni correction).
